# Genetic characterization of seasonal influenza A (H3N2) viruses in Ontario during 2010–2011 influenza season: high prevalence of mutations at antigenic sites

**DOI:** 10.1111/irv.12219

**Published:** 2013-12-06

**Authors:** AliReza Eshaghi, Venkata R Duvvuri, Aimin Li, Samir N Patel, Nathalie Bastien, Yan Li, Donald E Low, Jonathan B Gubbay

**Affiliations:** a Ontario Agency for Health Protection and PromotionToronto, ON, Canada; b Mount Sinai HospitalToronto, ON, Canada; c Centre for Disease Modelling, York UniversityToronto, ON, Canada; d University of TorontoToronto, ON, Canada; e National Microbiology Laboratory, Public Health Agency of CanadaWinnipeg, MB, Canada; f The Hospital for Sick ChildrenToronto, ON, Canada

**Keywords:** Antigenic site mutations, genetic and antigenic characterization, phylogenetic analysis, positive selection analysis, seasonal influenza A (H3N2) virus

## Abstract

**Background:**

The direct effect of antigenic site mutations in influenza viruses on antigenic drift and vaccine effectiveness is poorly understood.

**Objective:**

To investigate the genetic and antigenic characteristics of human influenza A (H3N2) viruses circulating in Ontario during the early 2010–2011 winter season.

**Study design:**

We sequenced the hemagglutinin (HA) and neuraminidase (NA) genes from 41 A(H3N2) viruses detected in nasopharyngeal specimens. Strain typing was performed by hemagglutination inhibition (HI) assay. Molecular and phylogenetic tree analyses were conducted.

**Results:**

HA and NA genes showed high similarity to the 2010–2011 vaccine strain, A/Perth/16/2009 (H3N2)-like virus (97·7–98·5% and 98·7–99·5% amino acid (AA) identity, respectively). Compared to A/Perth/16/2009 strain, HA gene mutations were documented at 28 different AA positions across all five H3 antigenic sites, with a range of 5–11 mutations in individual viruses. Thirty-six (88%) viruses had 8 AA substitutions in common; none of these had reduced HI titer. Among Ontario isolates, 11 antigenic site AAs were positively selected with an increase in glycosylation sites.

**Conclusion:**

The presence of antigenic site mutations with high frequency among 2010–2011 influenza H3N2 isolates confirms ongoing adaptive H3N2 evolution. These may represent early phylogenetic changes that could cause antigenic drift with further mutations. Clinical relevance of antigenic site mutations not causing drift in HI assays is unknown and requires further investigation. In addition, viral sequencing information will assist with vaccine strain planning and may facilitate early detection of vaccine escape.

## Introduction

Influenza viruses are considered one of the most common causes of respiratory infection among humans.^1^,^2^ Although all age groups are infected by influenza viruses, most of the influenza-related hospitalizations occur in young children (<5 years of age) and in the elderly (>65 years of age), and most deaths are reported in the elderly.^3^ Subtypes of influenza A viruses (IAV) are distinguished based on the unique antigenic properties of the two surface glycoproteins, hemagglutinin (HA) and neuraminidase (NA).^4^

Hemagglutinin is known to be a major target region of neutralizing antibodies which inhibit binding with sialic acid receptors effectively.^5^ Mutations in the HA antigenic sites, designated A, B, C, D, and E in IAV of H3N2 subtype, may result in strains which can escape recognition by pre-existing neutralizing antibodies.^7^–^9^ Gradual accumulation of mutations at these sites is noted to be an integral component of evolutionary dynamics and impacts viral survival and fitness. This evolutionary mechanism, known as antigenic drift, is the basis for frequent updating of the composition of seasonal influenza vaccines.^6^ Antigenic drift variants of H3 viruses occur often, and these tend to replace older ones.^7^ The higher rate of amino acid (AA) substitutions (0·0097 per site per year) of H3 HA when compared to H1 HA (0·0058 per year per site) supports their higher evolutionary rates.^8^ Previous studies proposed that an antigenic drift variant of epidemiological importance usually requires simultaneous accumulation of at least four AAs across two or more antigenic sites of A to E.^9^–^11^ The current study reports the HA genetic and antigenic relatedness between H3N2 viruses circulating during August 2010 to January 2011 in Ontario and A/Perth/16/2009(H3N2)-like virus (A/Perth/16/2009), which was recommended as the H3N2 component of the 2010–2011. Further, we extended this study to understand the mutational trends at antigenic sites of global IAV (H3N2) isolates from 2010–2011, which were grouped by continent.

## Methods

### Specimen collection, RT-PCR and sequencing

Forty-one H3N2-positive samples were included in this study, which consisted of all early season samples and a random selection from later in the season. Real-time reverse transcription PCR (rRT-PCR) for influenza A and B was performed as an initial screen on samples from hospital (admitted patients only) and outbreak settings. Samples from ambulatory patients (office settings and emergency patients not admitted) underwent viral culture for respiratory viruses using rhesus monkey kidney cells [(RMK) (Diagnostic Hybrids, Inc., Athens, OH, USA)]. Following total nucleic acid extraction, overlapping primers were used to amplify four and three fragments encompassing the coding region of HA and neuraminidase (NA) gene, respectively^12^; amplicons subsequently underwent Sanger sequencing.

### Antigenic characterization

A representative subset of Ontario's isolates was submitted to Canada's National Microbiology Laboratory for strain characterization by hemagglutination inhibition (HI) assay. This was performed using 4 hemagglutination units of virus, 0·5% v/v turkey red blood cells and post-infection ferret antisera against A/Perth/16/2009. HI titers were defined as the reciprocal of the highest dilution of serum that completely inhibited hemagglutination of 0·5% v/v turkey red blood cells; an eightfold reduction in titer compared to the reference strain was considered significant.^21^

### Sequence data collection and usage

In addition to 41 HA sequences obtained from Ontario's isolates, the analysis was performed with an enhanced dataset. A total of 1072 HA protein sequences of H3N2 (August 2010–January 2011) viruses of different circulating strains from Africa (73), Asia (372), Europe (133), Oceania (85), South America (60) North America (349; Canada excluded), and Canada (67; Ontario excluded) were downloaded from the GISAID (the Global Initiative on Sharing All Influenza Data) database.

HA and NA study sequences have been submitted to GenBank under accession numbers JQ658888 to JQ658927 and JQ658928 to JQ658967, respectively.

### Genetic characterization and phylogenetic analysis

HA1 sequence assembly was carried out using Vector NTI ContigExpress (Life Technologies™, Carlsbad, CA, USA). BioEdit was used for raw sequence curation and for multiple alignments of protein and nucleotide sequences.

A HA1 phylogenetic tree of Ontario's strains and a selection of global sequences were generated with Molecular Evolutionary Genetic Analysis (mega 4.0) using the neighbor-joining method algorithm.^13^ Evolutionary distance was computed using the maximum composite likelihood method. Statistical significance of the tree topology was tested by bootstrap analysis of 1000 pseudoreplicate datasets.^13^ The tree was visualized using dendroscope version 2.2.1.^14^

### Estimation of positive selection

To assess whether Ontario HA1 of H3N2 virus underwent positive selection when compared to MDCK-grown A/Perth/16/2009 (GenBank accession # GQ293081.1), we assessed the site-specific dN/dS and likelihood-ratio tests (LRT) using phylogenetic analysis using maximum likelihood (PAML 4.4 version).^15^ Bayes empirical Bayes (BEB) approach (implemented in CODEML) was used to calculate the posterior probabilities, ‘pp’ (taking sampling errors into account) of the inferred positively selected sites (PSS). Sites with high pp coming from the class with dN/dS> 1 (*P* > 95%) are inferred to be under positive selection.

### Prediction of glycosylation sites

Potential N-linked glycosylation sites were predicted using NetNGlyc 1.0 Server.^16^ A threshold value of >0·5 average potential predicts glycosylation sites.

### Structural modeling

Three-dimensional structures of the HA proteins of representative Ontario isolates were compared with A/Perth/16/2009 and constructed using related crystal structures (A/Aichi/2/1968; Protein Data Bank accession number 3HMG) using pymol.^17^

### Ethics Statement

This study was exempt from The University of Toronto's Health Sciences Research Ethics Board review as it involved deidentified respiratory tract samples that were tested as part of a clinical virology service provided by Public Health Ontario Laboratories. All test-positive samples and a proportion of test-negative samples are stored for possible further laboratory-based surveillance work. Samples and isolates included in this study were analyzed as part of the routine respiratory viral molecular surveillance program that supports Ontario's Ministry of Health and Long-Term Care.

## Results

### Sequence and phylogenetic analysis

Complete HA and NA sequences of the 41 Ontario H3N2 samples were analyzed and compared against vaccine strains and were shown to be most closely related to the 2010–2011 seasonal vaccine H3N2 strain, A/Perth/16/2009, with 97·8–99·0% and 98·0–98·7% identity at the nucleotide level, respectively. Identity at the AA level was 97·7–98·5% and 98·7–99·5%, respectively.

Comparative analysis with A/Perth/16/2009 revealed a mean of 8·7 (range 5–11) mutations across all antigenic sites. Thirty-six (88%) Ontario isolates had eight AA antigenic site substitutions in common. These included K144N at antigenic site A, D53N and E280A at site C, T212A, S214I, and I230V at site D, and K62E and Y94H at site E. In addition, 15 (37%) Ontario isolates had a unique substitution of isoleucine by methionine at residue 140 in antigenic site A, not observed among other Canadian or global isolates. Several other mutations at antigenic sites were observed among Ontario's strains such as T48A, E50K/G, N312S, and A304T (site C); S146G and M168I (site A); R208K V213A, I242K (site D); I260M, R261Q, Q75H, and K92R (site E); and S199A, F159Y, and I192T (site B). Mutations outside of antigenic sites also documented were as follows: T10P, P162S, R220K, N225D, D291E, and V323I. AA residues at the HA receptor binding sites remained conserved among all isolates except two, A/Ontario/C728447/2011 and A/Ontario/C76206/2011, harboring N133D and N225D, respectively (Figure S1). When the HA of Ontario's isolates was compared to A/Victoria/361/2011, the 2012–2013 vaccine strain (GISAID isolate id # EPI_ISL_101506), they showed an identity of 97·8–99% and 97·2–98·7% at the nucleotide and AA levels, respectively.

**Figure 1:**
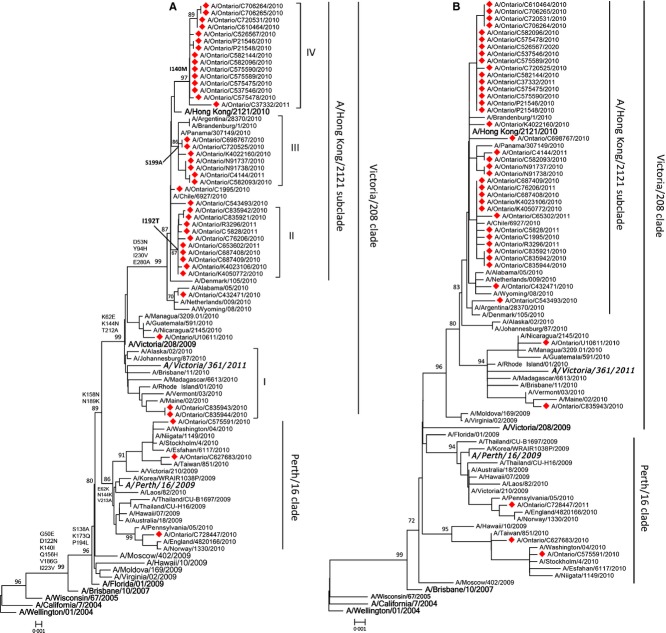
Phylogenetic relationship of full-length HA (A) and NA (B) sequences of influenza A(H3N2) virus isolates identified in Ontario during 2010–2011 influenza season. Multiple sequences alignment and phylogenetic trees were constructed using Clustal W and neighbor-joining algorithm running within mega 5.05 software. Tree topology was supported by bootstrap analysis with 1000 pseudo-replicate datasets. Bootstrap values >70% are included for key nodes. The scale bar represents the number of nucleotide substitutions per site between close relatives. Viruses characterized in the present study are marked with a filled diamond. Reference strains are in bold face. Vaccine strains for 2009–2012 (A/Perth/16/2009) and 2012–2013 (A/Victoria/361/2011-like virus) are bolded and italicized. Signature AA changes in HA tree are annotated at the nodes of each cluster.

**Figure 2:**
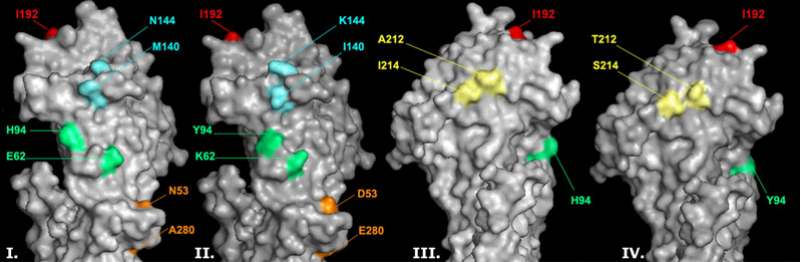
Effect of hemagglutinin protein antigenic site mutations on protein structure. An Ontario representative isolate detected during the 2010–11 influenza season is compared to the A/Perth/16/2009(H3N2)-like virus. Three-dimensional models of the H3 HA molecules of A/Ontario/C706264/2010(H3N2) (I and III) and A/Perth/16/2009(H3N2) were constructed based on the HA crystal structure of A/Aichi/2/1968 (Protein Data Bank code: 2HMG). The isoleucine at position 192 in antigenic site B, which has not mutated from the wild type in this isolate, is shown in red. Mutations in antigenic sites A (K144N, I140M; cyan), C (D53N, E280A; orange), D (T212A, S214T; yellow), and E (K62E, Y94H; green) are also shown. The structures are presented using pymol. For sequence alignment of A/Ontario/C706264/2010(H3N2) to A/Perth/16/2009(H3N2), see Figure S2.

The HA phylogenetic tree showed that 38 (93%) isolates fell within the recently emerged Victoria/208/2009 clade, and the remaining 3 (7%) belong to the Perth/16/2009 clade (Figure 1). Four recognizable genetic groups were identified among Ontario's strains that were placed within the Victoria/208/2009 clade due to the presence of specific non-synonymous substitutions: I. N312S (three isolates, 7%), II. I192T (10 isolates, 24%), III. S199A (seven isolates, 17%), IV. I140V (15 isolates, 37%). Three of these genetic groups (II, III, and IV; 86% of Ontario isolates) were clustered with the newly emerged subclade, A/Hong Kong/2121/2010, defined by D53N, Y94H, I230V, and E280A within the Victoria/208/2009 clade.^18^ The HA protein structure of a representative Ontario isolate demonstrates conformational changes in antigenic sites as a result of the point mutations detected (Figure 2 and Figure S2).

The NA gene phylogenetic tree showed the same distribution pattern as HA. Compared with the NA gene of A/Perth/16/2009, 38 (92%) of Ontario's isolates carried the substitution of serine (S) by asparagine (N) at position 367 (S367N), lysine (K) by threonine (T) at position 369 (K369T), and isoleucine (I) by leucine (L) at position 464 (I464L). Less frequent non-synonymous mutations that include E41N, M34V I62M, D127N, T138A, I176V, R210K, I307M, L338F, N342D, I381M, N402D, K415R, and R430S were observed among 2–4% of isolates. No mutations were observed at any of eight catalytic residues, which contact the NA inhibitor, oseltamivir, directly or among the 11 framework residues responsible for stabilizing the active binding site.^19^,^20^

### Glycosylation patterns

The nine putative N-glycosylation sites (Asn-Xaa-Ser/Thr) predicted at AA positions 8, 22, 38, 63, 126, 133, 165, 246, and 285 in A/Perth/16/2009 were identified in all of Ontario's 2010–2011 isolates. In addition, 38 (93%) isolates were shown to possess an additional glycosylation site (NNS) due to a K144N substitution. This K144N substitution was also observed among 72·2% of global isolates [range 42% to 97% of global isolates (Table S2)].

### Antigenic analysis

Eighteen representative H3N2 isolates were submitted for HI assay; all were antigenically related to the A/Perth/16/2009. Only one strain, A/Ontario/U10611/2010, showed eightfold reduction in HI titer (HI titer 80) when compared with A/Perth/16/2009 (HI titer 640). This strain exhibited four mutations at antigenic sites A (AA 144), D (AA 212 and 214), and E (AA 62). Three additional HA mutations, D291, K468T, and D489N, were exclusive to this isolate; there were no significant neuraminidase mutations.

The remaining 17 isolates were antigenically similar to A/Perth/16/2009. Eleven percent and 17% of isolates exhibited non-significant fourfold and twofold reductions, respectively. Three of the 10 isolates with a mutation (I192T) at antigenic site B were tested by HI; all showed non-significant twofold to fourfold HI titer reductions. The remaining seven I192T isolates were not of adequate HI titer to characterize. No correlation was observed between the presence of 10AA mutations across four antigenic sites (A, C, D and E) and HI titers (Table 1).

**Table 1:** Hemagglutination inhibition antibody titers and antigenic site mutations in influenza A(H3N2) isolates observed in Ontario, Canada, between November 2010 and February 2011

				Mutations occurred at antigenic sites (positively selected sites)													
	HI assay titers			Site A		Site B		Site C		Site D			Site E				
									
Ontario isolates	Specimen	RA^#^	Sp/RA#	140	144	192	199	53	280	212	214	230	62	94	260	261	No. of mutations
A/Ontario/C575591/10	640	320	2	–	–	–	–	–	–	–	I	–	–	–	M	Q	3
A/Ontario/C627683/10	320	640	1/2	–	–	–	–	–	A	–	I	–	–	–	M	Q	4
A/Ontario/U10611/10	80	640	1/8	–	N	–	–	–	–	A	I	–	E	–	–	–	4
A/Ontario/C543493/10	320	320	1	–	N	–	–	N	A	A	I	V	E	H	–	–	8
A/Ontario/C1995/10	80	160	1/2	–	N	–	–	N	A	A	I	V	E	H	–	–	8
A/Ontario/C575478/10	320	320	1	M	N	–	–	N	A	A	I	V	E	H	–	–	9
A/Ontario/C582096/10	320	320	1	M	N	–	–	N	A	A	I	V	E	H	–	–	9
A/Ontario/P21548/10	320	320	1	M	N	–	–	N	A	A	I	V	E	H	–	–	9
A/Ontario/C706264/10	640	640	1	M	N	–	–	N	A	A	I	V	E	H	–	–	9
A/Ontario/C582093/10	640	320	2	–	N	–	A	N	A	A	I	V	E	H	–	–	9
A/Ontario/C720531/10	640	320	2	M	N	–	–	N	A	A	I	V	E	H	–	–	9
A/Ontario/C537546/10	640	320	2	M	N	–	–	N	A	A	I	V	E	H	–	–	9
A/Ontario/C526567/10	320	160	2	M	N	–	–	N	A	A	I	V	E	H	–	–	9
A/Ontario/C653602/11	640	1280	1/2	–	N	T	–	N	A	A	I	V	E	H	–	–	9
A/Ontario/R3296/11	320	1280	1/2	–	N	T	–	N	A	A	I	V	E	H	–	–	9
A/Ontario/C76206/11	320	1280	1/4	–	N	T	–	N	A	A	I	V	E	H	–	–	9
A/Ontario/C582144/10	320	320	1	M	N	–	A	N	A	A	I	V	E	H	–	–	10

RA^#^, Reference antigen: A/Perth/16/09; Sp/RA#, Specimen/Reference antigen ratio.

### Evidence for positive selection

The average dN/dS ranged from 0·149 to 0·340 among all codon substitution models (Table S1). Both the M8 and M2a models suggested the presence of PSS with a proportion ranging from 4·0% (p1 = 0·040 with ω2 = 6·144) to 3·3% (p2 = 0·033 with ω2 = 6·839). A total of eleven PSS, 53, 62, 94, 140, 144, 192, 199, 212, 214, 230, and 280, were observed with posterior probability >50% (Table S1). All 11 mutations were observed across the four HA antigenic sites, of which seven substitutions (K144N in site A; D53N and E280A in site C; T212A and S214I in site D; K62E and Y94H in site E) were observed among 85–100% of Ontario isolates and substitution I140M in 37% of the Ontario isolates (Figure S1).

### Mutational patterns in global isolates

Ontario's isolates showed higher mutational frequency at positions I140M (present in 15/41, 37%) and I192T (present in 10/41, 24%) when compared with global isolates; these mutations were only present in 11 (0·96%) and 37 (3·2%) global isolates analyzed, respectively. Five (positions 144, 280, 212, 214, and 62) of 11 positively selected sites were conserved in the isolates from the Americas (North and South America). The only substitution conserved among all global isolates irrespective of their origin was S214I (Table S2).

## Discussion

In this study, we have characterized seasonal clusters of Influenza A(H3N2) circulating during the 2010–2011 winter season in Ontario. Nucleotide analysis of the complete coding region of these isolates showed close similarity to the vaccine strain, A/Perth/16/2009. HA nucleotide identity was higher than the NA identity when compared with A/Perth/16/2009. However, HA had a lower AA identity with the A/Perth/16/2009. This implies that nucleotide mutations in HA are more likely to be non-synonymous than those occurring in NA, in keeping with the propensity of this protein to evolve at a higher rate.^8^ Mutations were documented at 28 different AA positions within H3 antigenic sites. Five to 11 antigenic site AA mutations were observed in individual isolates (Figure S1).

Co-circulation of variants distinguished by specific AA substitutions was seen among Ontario's H3N2 strain. Phylogenetic trees of the HA and NA genes show that Ontario's 2010–11 isolates fell into two distinct genetic clades represented by A/Perth/16/2009 and A/Victoria/208/2009, with the majority (93%) belonging to Victoria 208 clade. Several phylogenetic subgroups within the A/Victoria/208/2009 clade were identified, with the majority (86%) of Ontario's isolates falling within the newly emerged subclade, A/Hong Kong/2121/2010. Due to the emergence of the Victoria 208 clade of A/Perth/16/2009 strain, WHO recommended a change to the H3N2 strain component of the 2012–2013 influenza vaccine, to A/Victoria/361/2011-like virus.^21^

Evolutionary analysis of the HA of Ontario's 2010–2011 H3N2 isolates revealed strong evolutionary selection pressure (dN/dS = 6·8), resulting in 11 PSS compared to A/Perth/16/2009 (Table S1). Three of the PSSs (53, 144, and 192) were reported as frequently changeable sites in H3N2 HA evolution and also observed in previously reported vaccine escape mutants.^22^,^23^ The mutation at AA 192 was observed among Canadian isolates (including Ontario isolates), but not other global isolates. AA 192 is located within the 190 helix, which contains receptor binding sites (RBS). It has been shown that certain AA alterations within the receptor binding regions can alter sialic acid receptor binding specificity. Although one study documented a shift in sialic acid receptor binding specificity from α-2,3 to α-2,6 glycans following a T192I change in H5N1, another publication did not show any change in receptor specificity as a result of generation of a I192T mutation on a wild-type H5N1 background.^24^,^25^ This mutation has not been evaluated in the setting of a human H3N2 background.

All of Ontario's 2010–2011 isolates possessed the same nine glycosylation sites identified in A/Perth/16/2009. Thirty-eight (93%) of Ontario's viruses were shown to possess an additional glycosylation site (NNS) due to a K144N substitution, which also resulted in an AA change in antigenic site A. This mutation was also common among global isolates (Table S2). A position 144 N-glycosylation site has previously been observed as an infrequent mutation in H3N2 evolution and reported to be involved in masking the key site for antigenic change in A/Fujian/411/02-like strains from the 2002–2003 influenza season.^26^ However, this mutation was not responsible for the antigenic drift from A/Moscow/10/99 to A/Fujian/411/02.^11^ We did not observe the N144D mutation in the Ontario H3N2 isolates, whereas it was present in European and African H3N2 isolates (2010–2011), documented in 13% and 35% of respective strains. AA substitutions, particularly in antigenic site A (140–146), were reported as a typical signature for antigenically distant viruses of epidemic significance.^5^

A substitution from isoleucine to methionine at position 140 was observed in 37% of Ontario's 2010–2011 isolates, but was only found in 0·96% of other global 2010 to 2011 isolates. I140M was previously detected in six A/Brisbane/10/2007-like H3N2 isolates collected in the USA in 2008 (accession no: ADY05342, ACD69148) and 2009 (ACT67814, ACT67819, ADK94334, and ADM26784). Substitutions D53N (in antigenic site C) and I140M (in antigenic site A) were found to decrease affinity of H3N2-specific antibodies based on microneutralization (MN) assay with A/Perth/16/2009 (H3N2) antiserum; however, their impact on immune recognition is not known.^27^

Despite the presence of up to 11 mutations at four antigenic sites, considerable evidence for antigenic drift was not observed based on the data obtained by HI assay. This is in contrast to the previously held idea that four mutations across two or more antigenic sites would predict a propensity for antigenic drift.^28^ Only one strain in our 2010–2011 collection has shown evidence of drift in the HI assay (eightfold difference with A/Perth/16/2009). Genome sequence and phylogenetic analysis of this isolate revealed highest homology to isolates from Central America. This was consistent with the patient history of recent travel to Honduras shortly before sampling (Figure 1). Interestingly, this isolate only has four substitutions at site A (144), site D (212, 214), and site E (62). Our findings emphasize the poor correlation between antigenic profile and AA substitutions in the HA protein of influenza viruses, as has been previously observed.^29^ During winter of 2010–2011, the emergence of >4 substitutions in three different H3 antigenic sites was observed in Quebec, Canada, suggesting antigenic drift. However, HI and MN results showed reduced titer in only one strain (eightfold difference in titers), while 19 others remained antigenically similar to A/Perth/16/2009, but exhibited titer differences (twofold to fourfold), below the standard definition of antigenic drift.^28^ Of interest, all three isolates carrying I192T HA mutation and of adequate HA titer for strain typing had a mild twofold to fourfold HI titer reduction. In addition, a five province Canadian study of influenza vaccine effectiveness in ambulatory patients found similar H3N2 HA mutations at antigenic sites without any changes in HA titers, along with a low vaccine effectiveness of 39% in an interim analysis during the early part of the 2012–13 influenza season.^30^

The majority of the antigenic site mutations identified in this study appear at the surface structures of antigenic sites that are accessible to antibodies and resulted in conformational changes (Figure 2). Such structural changes at antigenic sites (epitopes) could possibly facilitative escape from neutralizing antibody. In addition, mutations K62E, Y94H, and K144N cause alteration in side chain charge. Moreover, mutations E280A, T212A, and S214I will change the polarity of these AAs.

In conclusion, the presence of up to 11 positively selected antigenic site mutations with high frequency among 2010–2011 influenza season H3N2 isolates confirms the ongoing adaptive evolution of circulating H3N2 strains. Further work is needed to better understand the clinical relevance of mutations at antigenic sites that do not result in drift in HI assays. In addition, further investigation is needed to better understand the extent to which viral sequencing should influence vaccine strain selection, even when there is no associated drift in HI assays. Relying on detection of antigenic drift in HI assays before altering candidate vaccine strains may not be sensitive enough to detect clinically relevant changes in vaccine effectiveness.

## Supporting Information

**Figure S1.** HA Variability among Ontario's influenza A (H3N2) virus during 2010–2011 Influenza season.

**Figure S2.** Amino acid sequence alignment of A/Ontario/C706264/2010(H3N2) to MDCK grown A/Perth/16/2009 (Genbank accession # GQ293081.1).

**Table S1.** Parameter estimates, dN/dS, values of log-Likelihood (l), positive selection sites, and Likelihood Ratio Tests (LRT) in the Hemagglutinin gene analysis of influenza A H3N2 viruses circulating in Ontario, Canada between November 2010 and February 2011.

**Table S2.** Comparative analysis of ON H3N2 HA mutational patterns at antigenic sites with other geographical regions.
