# Supplementary figures and images for: Genetic characterization of seasonal influenza A (H3N2) viruses in Ontario during 2010–2011 influenza season: high prevalence of mutations at antigenic sites
Source: Influenza Other Respir Viruses. 2013 Dec 6;8(2):250–7. doi: 10.1111/irv.12219 (PMC4186474; doi:10.1111/irv.12219)

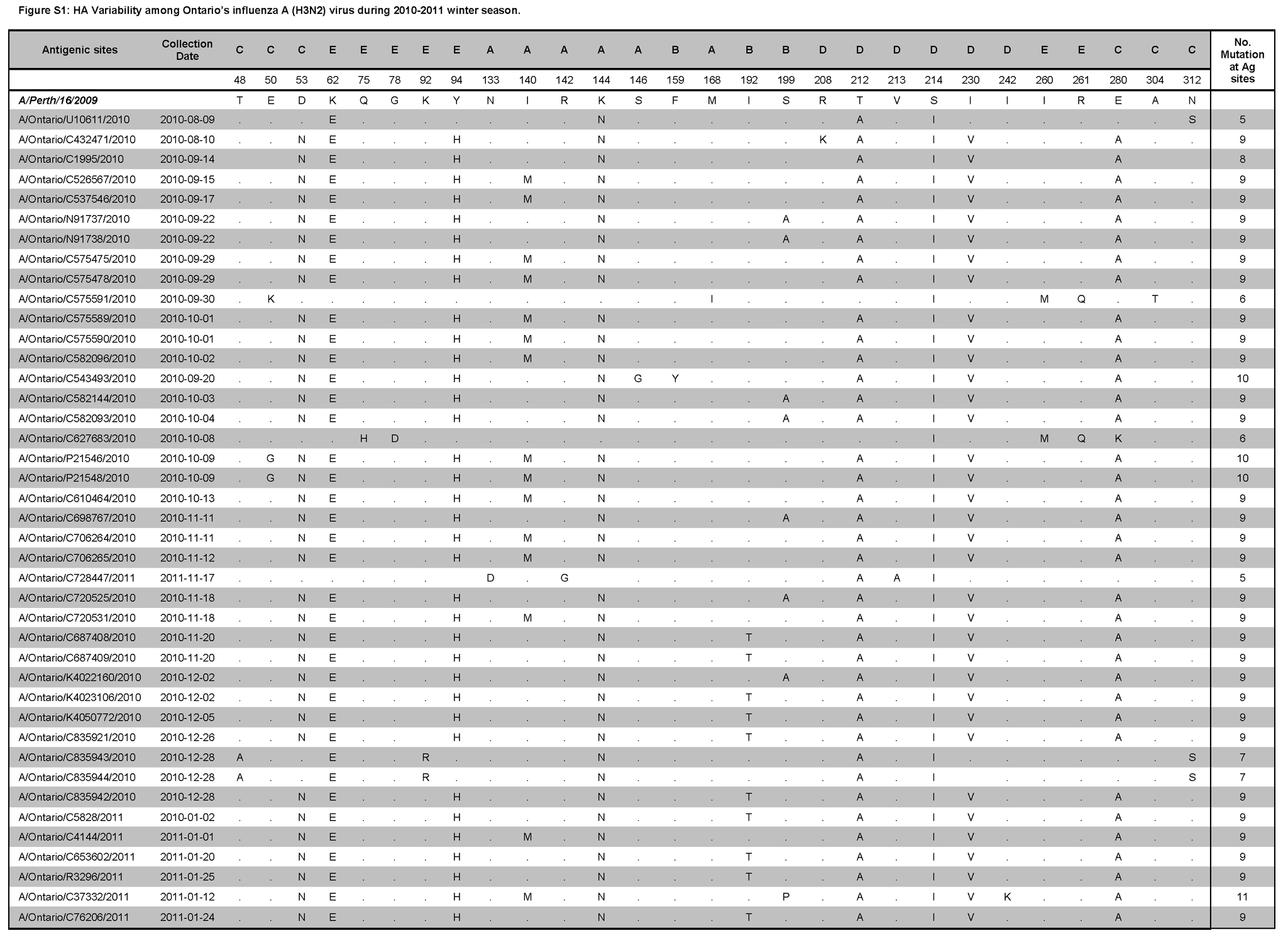

Supplement: Supplementary file 1 — Figure S1. HA Variability among Ontario's influenza A (H3N2) virus during 2010–2011 Influenza season. [file irv0008-0250-SD1.tif]

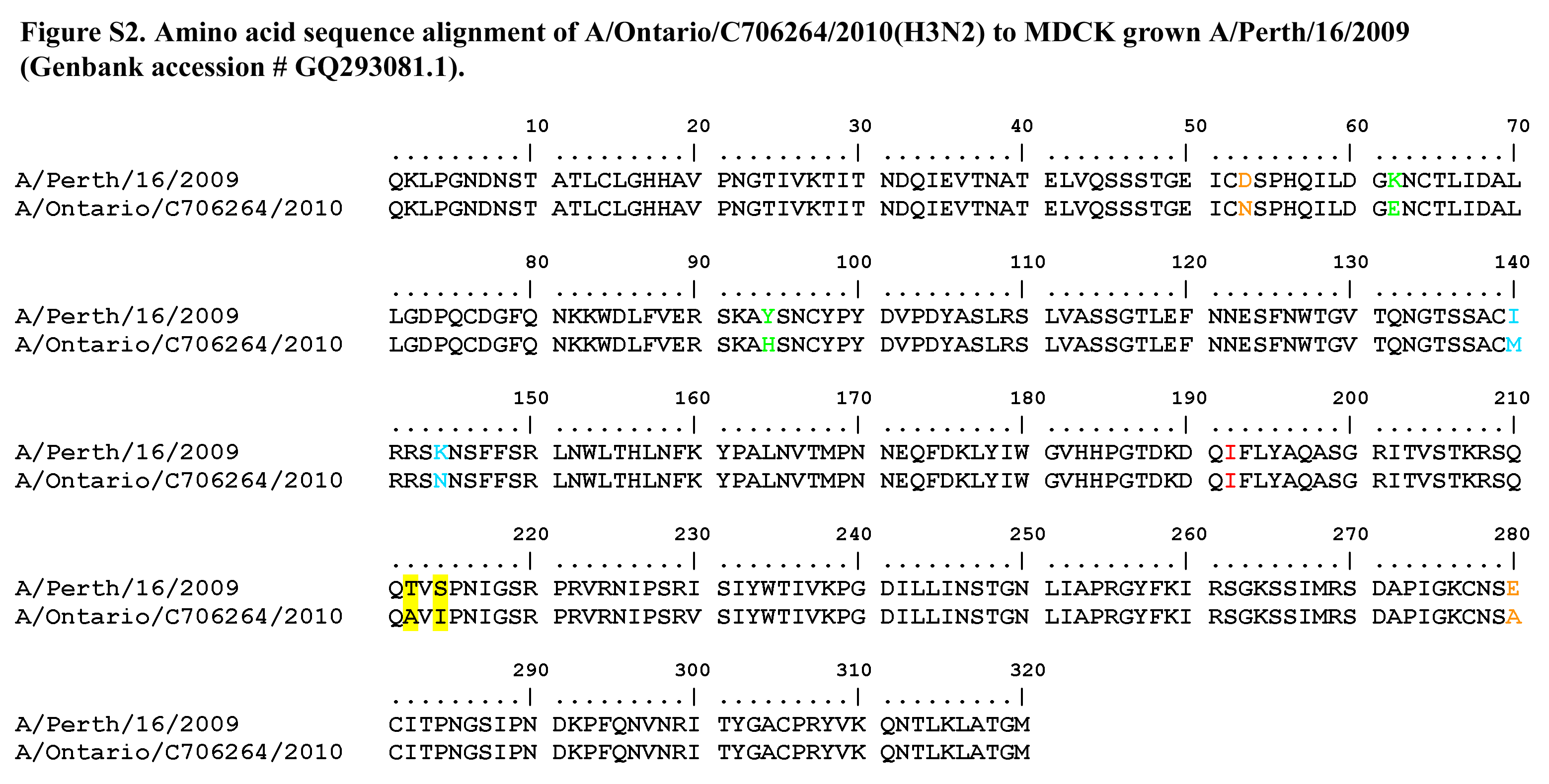

Supplement: Supplementary file 2 — Figure S2. Amino acid sequence alignment of A/Ontario/C706264/2010(H3N2) to MDCK grown A/Perth/16/2009 (Genbank accession # GQ293081.1). [file irv0008-0250-SD2.tif]
