# Supplementary material for: Genetic characterization of seasonal influenza A (H3N2) viruses in Ontario during 2010–2011 influenza season: high prevalence of mutations at antigenic sites
Source: Influenza Other Respir Viruses. 2013 Dec 6;8(2):250–7. doi: 10.1111/irv.12219 (PMC4186474; doi:10.1111/irv.12219)
Supplement: Supplementary file 3 — Table S1. Parameter estimates, dN/dS, values of log-Likelihood (l), positive selection sites, and Likelihood Ratio Tests (LRT) in the Hemagglutinin gene analysis of influenza A H3N2 viruses circulating in Ontario, Canada between November 2010 and February 2011. [file irv0008-0250-SD3.docx]

**Supplementary Figure Legends**

**Figure S1. HA Variability among Ontario’s influenza A (H3N2) virus during 2010-2011 Influenza season.**

Antigenic site amino acid (AA) changes in HA1 proteins of representative influenza A (H3N2) isolates detected in Ontario during 2010-2011 winter season with respect to vaccine strain, A/Perth/16/2009. Vaccine strain is indicated in boldface. Dots represent AAs similar to the consensus. The AA residues mapped at previously defined antigenic sites are indicated by letters A-E above relevant residue.

**Figure S2. Amino acid sequence alignment of A/Ontario/C706264/2010(H3N2) to MDCK grown A/Perth/16/2009 (Genbank accession # GQ293081.1).**
