# Supplementary material for: Genetic characterization of seasonal influenza A (H3N2) viruses in Ontario during 2010–2011 influenza season: high prevalence of mutations at antigenic sites
Source: Influenza Other Respir Viruses. 2013 Dec 6;8(2):250–7. doi: 10.1111/irv.12219 (PMC4186474; doi:10.1111/irv.12219)
Supplement: Supplementary file 4 — Table S2. Comparative analysis of ON H3N2 HA mutational patterns at antigenic sites with other geographical regions. [file irv0008-0250-SD4.docx]

**Supplementary Table S1.** Parameter estimates, dN/dS, values of log-Likelihood (l), positive selection sites, and Likelihood Ratio Tests (LRT) in the Hemagglutinin gene analysis of influenza A H3N2 viruses circulating in Ontario, Canada between November 2010 and February 2011.

| **Model** | **Parameter estimates** | **dN/dS** | **Log-likelihood(l)** | **PSS*^a^*** | **Model comparison (2Δ*l*, d.f, p)** |
| --- | --- | --- | --- | --- | --- |
| M1 (neutral) | ω0 =0.021, ω1 =1 | 0.149 | -2382.96 | Not allowed | M1a vs. M2a: 69.09 d.f = 2**,** p < 0.0001 |
|  | p0 =0.868 |  |  |  |  |
|  | (p1 =0.131) |  |  |  |  |
| M2 (selection) | ω0 = 0.056, ω1 =1 | 0.338 | -2348.43 | **140** (A) |  |
|  | **ω2= 6.839** |  |  | **192, 199** (B) |  |
|  | p0=0.90, p1=0.058 |  |  | 53, **280** (C) |  |
|  | **(p2 = 0.033)** |  |  | **212,214,** 230 (D) |  |
|  |  |  |  | **94** (E) |  |
| M7 (beta) | p=0.005, q=0.008 | 0.333 | -2394.49 | Not allowed | M7 vs. M8: 90.54 d.f = 2, p < 0.0001 |
|  |  |  |  |  |  |
| M8 (beta + ω) | p0=0.959 | 0.34 | -2349.22 | **140**, 144 (A) |  |
|  | **(p1= 0.040)** |  |  | **192, 199** (B) |  |
|  | p=0.183, q=1.332 |  |  | **53, 280** (C) |  |
|  | **ω2 = 6.144** |  |  | **212, 214, 230** (D) |  |
|  |  |  |  | 62, **94** (E) |  |

Neutral models (M1a, and M7) were compared with their respective alternative (selection) models (M2a and M8), which allow *ω* > 1. Model comparison can be calculated using 2Δ*l* = 2 (*l*_1_ - *l*­_0_)), where *l*_1 =_ LRT of alternative model; and *l*­_0 =_ LRT of null model. Proportion of PSS and their corresponding *ω*-values in M2a and M8 models are in bolded italics. The significant **P** values indicated that all analyses find very strong evidence for the selection model. A, B, C, D, and E are antigenic sites of HA.

*a* PSS: Positively selected sites using BEB analysis [28]. Posterior probability (pp) of PSS of M2a model: 90% to 98% (53, 230); and ≥99% (94, 140, 192, 199, 212, 214, and 280); pp of PSS of M8 model: 90% to 98% (62, 144); and ≥99% (53, 94, 140, 192, 199, 212, 214, 230, and 280). Bold AA sites: pp ≥99%; Underlined AA sites: pp ≥90% to ≤98%

| **Supplementary Table S2: Comparative analysis of ON H3N2 HA mutational patterns at antigenic sites with other geographical regions.** | | | | | | | | |
| --- | --- | --- | --- | --- | --- | --- | --- | --- |
|  |  |  |  |  |  |  |  |  |
|  |  |  |  |  |  |  |  |  |
| **Mutation** | **Mutation frequency (actual number of mutants)** | | | | | | | |
|  | **Ontario**  **(n=41)** | **Canada**  **(n = 67)** | **North America**  **(Canada excluded)**  **(n= 349)** | **South**  **America**  **(n = 60)** | **Asia**  **(n=372)** | **Europe**  **(n=133)** | **Africa**  **(n=73)** | **Oceania**  **(n= 85)** |
| **I140M** | 37% (15) | 0% (0) | 1% (5) | 0% (0) | 1% (5) | 0% (0) | 0% (0) | 1% (1) |
| **K144N** | 93% (38) | 97% (65) | 91% (318) | 95% (57) | 42% (157) | 74% (98) | 64% (47) | 95% (81) |
| **N144D** | 0% (0) | 0% (0) | 0.28% (1) | 0% (0) | 0.53% (2) | 13% (20) | 35% (26) | 0% (0) |
| **I192T** | 24% (10) | 28% (19) | 1% (5) | 0% (0) | 3% (10) | 2% (3) | 0% (0) | 0% (0) |
| **S199A** | 17% (7) | 15% (10) | 28% (99) | 58% (35) | 0% (1) | 15% (20) | 0% (0) | 2% (2) |
| **D53N** | 85% (35) | 67% (42) | 30% (106) | 85% (51) | 18% (67) | 54% (72) | 14% (10) | 54% (46) |
| **E280A** | 88% (36) | 80% (53) | 64% (223) | 83% (50) | 31% (117) | 43% (57) | 10% (7) | 52% (44) |
| **T212A** | 95% (39) | 99% (66) | 97% (340) | 95% (57) | 57% (212) | 89% (119) | 1% (1) | 91% (77) |
| **S214I** | 100% (41) | 99% (66) | 99% (344) | 100% (60) | 100% (372) | 100% (133) | 100% (73) | 100% (85) |
| **I230V** | 85% (35) | 80% (53) | 66% (232) | 80% (48) | 40% (149) | 50% (67) | 11% (8) | 52% (44) |
| **K62E** | 93% (38) | 97% (65) | 91% (319) | 93% (56) | 57% (213) | 85% (113) | 1% (1) | 95% (81) |
| **Y94H** | 85% (35) | 78% (52) | 67% (234) | 83% (50) | 40% (150) | 51% (68) | 11% (8) | 95% (81) |
| **I260M** | 5% (2) | 2% (1) | 1% (5) | 2% (1) | 40% (150) | 10% (13) | 7% (5) | 5% (4) |
| **R261Q** | 5% (2) | 3% (2) | 2% (6) | 2% (1) | 58% (216) | 10% (13) | 1% (1) | 5% (4) |
